# Cardiac Rehabilitation After Mitral Valve Intervention: Tailored Assessment, Management, and Exercise Training

**DOI:** 10.3390/jcdd12070265

**Published:** 2025-07-09

**Authors:** Francesco Perone, Monica Loguercio, Federica Sabato, Annalisa Pasquini, Marina Ostojic, Ashot Avagimyan, Vaida Sileikiene, Joanna Popiolek-Kalisz, Aneta Aleksova, Marco Ambrosetti

**Affiliations:** 1 Cardiac Rehabilitation Unit, Rehabilitation Clinic ‘Villa delle Magnolie’, Castel Morrone, 81020 Caserta, Italy; 2 Cardiovascular Rehabilitation Unit, ASST Crema, Santa Marta Hospital, 26027 Rivolta D’Adda, Italy; msloguercio@gmail.com (M.L.); marco.ambrosetti@asst-crema.it (M.A.); 3 Clinica Hildebrand Rehabilitation Center Brissago, Via Crodolo, 6614 Brissago, Switzerland; federicasabato1@gmail.com; 4 Department of Cardiovascular Medicine, Fondazione Policlinico Universitario A. Gemelli, IRCCS, Largo Agostino Gemelli 8, 00168 Rome, Italy; dott.annalisapasquini@gmail.com; 5 Cardiology Clinic, University Clinical Center of Serbia, 11000 Belgrade, Serbia; drmarinaostojic@gmail.com; 6 Department of Internal Diseases Propedeutics, Yerevan State Medical University After M. Heratsi, Korun Street 2a, Yerevan 0025, Armenia; dr.ashotavagimyan@gmail.com; 7 Clinic of Cardiac and Vascular Diseases, Faculty of Medicine, Vilnius University, Ciurlionio Str. 21, LT-03101 Vilnius, Lithuania; sileikiene.vaida@gmail.com; 8 Vilnius University Hospital Santaros Clinics, Santariskiu Str. 2, Vilnius, LT-03101 Vilnius, Lithuania; 9 Department of Clinical Dietetics, Medical University of Lublin, ul. Chodzki 7, 20-093 Lublin, Poland; popiolekkalisz@gmail.com; 10 Department of Cardiology, Cardinal Wyszynski Hospital in Lublin, Al. Krasnicka 100, 20-718 Lublin, Poland; 11 Cardiothoracovascular Department, Azienda Sanitaria Universitaria Giuliano Isontina, 34139 Trieste, Italy; aaleksova@units.it; 12 Department of Medical, Surgical and Health Sciences, University of Trieste, 34139 Trieste, Italy

**Keywords:** cardiac rehabilitation, mitral valve intervention, physical activity, mitral valve surgery, mitral valve repair, transcatheter edge-to-edge repair

## Abstract

Cardiac rehabilitation should be suggested after mitral valve intervention. Physical exercise is associated with improved cardiorespiratory fitness and clinical outcome and reduced rehospitalization and mortality in patients after heart valve surgery. Tailored assessment is the first step before starting a cardiac rehabilitation program. Physical examination, electrocardiogram, echocardiography, and peak exercise capacity stratify the risk of these patients when prescribing appropriate supervised aerobic and resistance exercise training. Cardiac rehabilitation participation impacts physical capacity, psychosocial function, and prognosis in patients after mitral valve surgery and transcatheter edge-to-edge repair. However, further evidence is needed on the efficacy and safety of cardiac rehabilitation programs, as well as standardization. In this review, we provide a contemporary and comprehensive update on the role of cardiac rehabilitation in patients after mitral valve intervention, after both mitral valve surgery and transcatheter mitral valve implantation. Specifically, we focus our review on the tailored assessment and management of these patients from post-operative to cardiac rehabilitation.

## 1. Introduction

Mitral regurgitation (MR) is a significant global health concern, affecting an estimated 24.2 million individuals worldwide. Among its various causes, mitral valve prolapse is the most prevalent, affecting 2–3% of the population. MR ranks as the second most common valvular disease in Europe. While there is a global increase in the incidence of mitral valve disease due to population aging and growth, age-standardized mortality rates have shown a decline [1,2].

MR is classified into two broad categories: primary and secondary MR. Secondary mitral regurgitation (SMR) arises from atrial or ventricular remodeling, whereas primary mitral regurgitation (PMR) can result from degenerative, rheumatic, or infectious etiologies. Degenerative causes, including fibroelastic deficiency and Barlow’s disease, are the most common in high-income countries, while rheumatic heart disease remains the predominant cause in low-income nations. The clinical presentation and management of PMR depend on the severity of the condition and the patient’s overall health status, underscoring the importance of early detection and appropriate intervention. In cases of severe acute MR, urgent surgical intervention is required. For symptomatic severe PMR, surgery is recommended when the surgical risk is deemed acceptable by a heart team. In asymptomatic individuals, the decision to intervene is typically based on echocardiographic assessments of the left ventricular ejection fraction (LVEF), left ventricular end-systolic diameter (LVESD), left atrial (LA) volume, and systolic pulmonary artery pressure (SPAP). Intervention thresholds include an LVEF of ≤60%, an LVESD ≥ 40 mm, LA volume exceeding 60 mL/m^2^, LA diameter > 55 mm, and SPAP > 50 mmHg [3]. In the absence of these criteria, vigilant monitoring is generally recommended, as premature intervention may pose more risks than benefits. Mitral valve repair remains the preferred procedure for valves without significant calcification or extensive prolapse. However, when repair is not feasible, mitral valve replacement, with the preservation of the subvalvular apparatus, is the next option to ensure long-term valve function. In patients with high surgical risk or those suitable based on echocardiographic findings, transcatheter edge-to-edge repair (TEER) can be considered as an alternative to traditional surgery [3]. TEER, derived from the Alfieri surgical technique, involves approximating and clipping the valve leaflets to reduce regurgitation. Initially, TEER raised concerns regarding potential stenosis, but evidence from the EVEREST II trial demonstrated its safety and efficacy, particularly in high-risk patients. This trial showed that TEER not only improved MR severity but also enhanced the New York Heart Association (NYHA) functional class and reduced left ventricular end-diastolic and systolic volumes. Additionally, TEER significantly decreased the mitral annular dimension in both functional and degenerative MR patients, with notable improvements in end-diastolic volume for functional MR [4]. Further supporting these findings, the MitraSwiss registry reported a procedural success rate of 91.5%, with favorable outcomes observed across various etiologies of MR, including both functional and degenerative forms. Mortality and major adverse cardiovascular event (MACE) rates were lower in degenerative MR patients, although functional MR patients, despite being younger, exhibited a higher burden of comorbidities. Importantly, the risk of procedural complications was minimal, with mortality rates below 1%. The etiology of MR did not correlate with poorer outcomes, suggesting that TEER can be a viable intervention for high-risk patients, regardless of MR subtype [5]. In addition to procedural outcomes, the prognostic significance of post-TEER changes in mitral valve gradients has been investigated. In PMR, increased mitral valve gradients after TEER are not independently associated with adverse events. However, a reduction in MR severity has been identified as a significant predictor of favorable clinical outcomes. Studies by Yoon et al. and Ludwig et al. emphasize that improving regurgitation severity, rather than managing post-procedural gradients, is the key to enhancing clinical outcomes [6,7].

Cardiac rehabilitation (CR) plays a critical role in the recovery and long-term health of patients with cardiovascular disease, reducing hospitalizations, improving cardiovascular risk profiles, and lowering mortality rates. Current guidelines recommend CR as a Class 1A intervention for cardiovascular disease patients, utilizing the FITT model (Frequency, Intensity, Time, Type) to optimize cardiovascular health. However, the landscape of rehabilitation following mitral valve interventions, particularly in PMR, remains less well-studied compared to rehabilitation after ischemic heart disease or heart failure [8]. Evidence regarding the safety and efficacy of high-intensity interval training in patients with valvular heart disease is still insufficient [9]. Regular physical activity is now a cornerstone of cardiovascular therapy, recommended in both elderly and younger patients with cardiovascular disease. Given that valvular heart disease affects 1–2% of young, exercising individuals, it is crucial to adhere to guidelines that promote safe participation in sports [10]. Therefore, this review aims to provide an overview of the available evidence regarding CR following the surgical or percutaneous treatment of PRM, summarize recommendations for developing an evidence-based rehabilitation program, and highlight the need for further research in this field.

## 2. Exercise Benefits in Patients After Mitral Valve Intervention

While the benefits of CR after coronary artery bypass graft (CABG) surgery have been well-established, evidence regarding the benefits of CR after valve surgery is less robust [11,12]. A systematic review and meta-analysis concluded that evidence regarding the impact of exercise-based CR on hospitalization, quality of life (QoL), and mortality in patients undergoing valve interventions remains inconclusive, primarily due to the low quality of available data [13].

**Figure 1:**
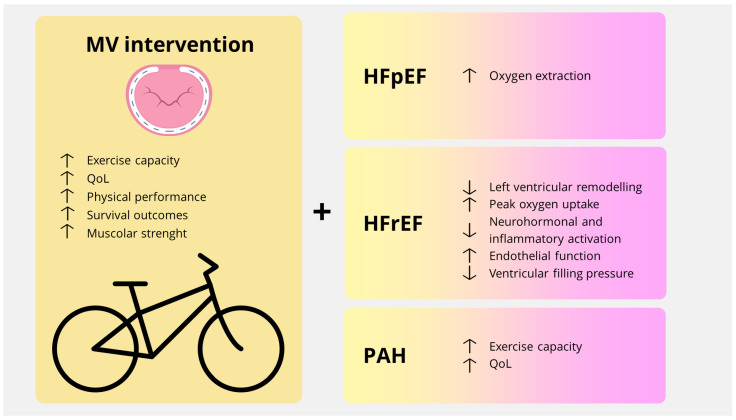
Benefits of physical activity in patients after mitral valve intervention. HFpEF, heart failure with preserved ejection fraction; HFrEF, heart failure with reduced ejection fraction; MV, mitral valve; PAH, pulmonary artery hypertension; QoL, quality of life.

However, comparisons between CABG and valve surgery patients have reported similar benefits in aerobic fitness for those participating in CR programs [14]. Data indicate increased exercise capacity and improved QoL with a minimal risk of adverse events in this population (Table 1) [15]. Although some authors argue that evidence is insufficient to support routine CR for individuals who have undergone valve surgery, they encourage regular exercise and a healthy lifestyle [16]. Current guidelines include these patients in CR programs [17,18]. This recommendation is supported by various data. Studies have shown that CR enhances exercise capacity, physical performance, survival outcomes, and muscular strength, leading to functional improvements within three months post-heart valve surgery or intervention. However, the additional benefits of incorporating resistance and balance training in this patient population remain inconclusive [19,20]. Racial and ethnic disparities remain in CR referrals, with Asian, Black, and Hispanic patients less likely to be enrolled after valve surgery. Furthermore, patients undergoing concomitant bypass surgery are more likely to be referred than those who undergo valvular intervention alone. CR enrolment has been associated with reduced hospitalization and mortality risk within the first year post-surgery [21]. When focusing on mitral valve interventions, stratification by procedure revealed a positive but non-significant effect on survival in patients undergoing CR after mitral valve surgery [22]. In comparison to patients recovering from aortic valve replacement, those recovering from mitral valve surgery exhibit worse exercise performance one-month post-surgery, although better results are seen in patients with MR than in those with mitral stenosis [23]. The approach to mitral intervention is also crucial. A study on minimally invasive mitral valve repair for severe organic MR, even in patients with minimal symptoms, found significant improvements in cardiopulmonary performance [24]. In particular, a transaxillary approach to mitral valve surgery has been linked to reduced postoperative recovery times, enabling earlier discharge without the need for extensive cardiopulmonary rehabilitation [25]. Regarding CR’s role in patients undergoing TEER, particularly elderly or multimorbid patients, less evidence is available. A case report involving a frail elderly patient undergoing CR pre- and post-TEER showed improvements in exercise endurance, handgrip strength, balance, and mobility, even after one year of follow-up [26]. Additionally, a retrospective analysis of 27 patients after MitraClip implantation indicated that inpatient CR was feasible, with benefits in improving daily living activities without significant complications [27]. Given that patients with mitral valve regurgitation may experience deterioration in ejection fraction, pulmonary hypertension, or heart failure with preserved ejection fraction (HFpEF), the effects of physical activity on these patients can be inferred from existing evidence. In patients with heart failure and reduced ejection fraction (HFrEF), exercise training has been shown to reverse left ventricular remodeling, improve aerobic capacity, and increase peak oxygen uptake (VO2), with moderate continuous training (MCT) being the most efficient, tolerated, and safe modality. Physical exercise has also been shown to reduce neurohormonal and inflammatory activation, improve endothelial function, and lower ventricular filling pressure in this population [28]. In patients with HFpEF, no significant changes in diastolic and systolic function, endothelial function, or arterial stiffness have been reported, but improvements in oxygen extraction, thanks to enhanced skeletal muscle oxygen extraction, have been preserved [29,30]. Patients with pulmonary artery hypertension can experience improvements in exercise capacity and QoL through exercise-based rehabilitation without increasing the mean pulmonary artery pressure [31]. Furthermore, patients discharged after valve heart interventions may experience increased inactivity, anxiety, and depression. Although a 2016 study showed significant improvements in peak VO2 within four months of CR participation in heart valve surgery patients, no improvements in mental health or exercise capacity were observed [32]. Nevertheless, physical activity can benefit mental health, even at levels below the recommended thresholds [33]. This highlights the importance of involving these patients in CR programs. While physical exercise after mitral valve surgery offers significant benefits, it also raises concerns regarding anticoagulation therapy and the risk of arrhythmias. For patients on anticoagulation therapy, guidelines recommend avoiding high-risk collision sports due to the increased risk of bleeding [18]. Regarding the risk of sudden cardiac death (SCD) after PMR intervention, it is important to note that SCD in patients with mitral valve prolapse (MVP) occurs at a rate of 0.2–0.4% per year, particularly in those with bileaflet prolapse, fibrosis in the papillary muscles, or mitral annulus disjunction (MAD). Arrhythmias in MVP are likely due to abnormalities in the mitral valve annulus, including fibrosis, hypertrophy, and mechanical stretch, which can trigger premature ventricular beats, with potential contributions from damaged Purkinje fibers. Late gadolinium enhancement in the papillary muscle region has been found to correlate with the presence of papillary muscle-based premature ventricular beats [34]. Following surgery, mechanical stretch is reduced, which alleviates arrhythmic events, particularly in younger patients without a long history of arrhythmias [35,36]. The European consensus suggests that, after valvular intervention, the same limitations applied to asymptomatic athletes with moderate native valve disease should apply, provided that pulmonary artery pressure and ventricular function remain unaffected [18]. Patients with MVP and mild to moderate regurgitation are allowed to participate in all physical activities, as long as there are no T-wave inversions in the inferior leads of the 12-lead electrocardiogram (ECG), no ventricular arrhythmias detected on a 24 h Holter monitor, and no family history of SCD [18]. Close monitoring with diagnostic tools such as 24 h Holter monitoring and exercise stress testing is essential to assess arrhythmias, hemodynamic responses, and functional capacity. In cases of syncope, T-wave inversion in inferior leads, or RBBB with superior axis ventricular extrasystoles, cardiac magnetic resonance should be considered. Aside from the severity of mitral insufficiency, it is crucial to assess ventricular volume and pulmonary pressure. Individuals with a LVESD ≤ 60 mm (or ≤35 mm/m^2^ in men, ≤40 mm/m^2^ in women), a LVEF > 60%, and resting pulmonary artery pressure below 50 mmHg are generally encouraged to participate in all sports after demonstrating good functional capacity during a maximal exercise test and the absence of concerning arrhythmias [18]. In conclusion, physical activity plays a significant role in improving exercise capacity and reducing the risk of mortality after heart valve surgery (Figure 1). Training can be safe for these patients when following guideline recommendations. However, further high-quality research is needed to fully understand the impact of rehabilitation programs, the specific components (such as resistance and balance training), and their long-term benefits for this patient population.

## 3. Patient Assessment and Management After Mitral Valve Intervention: Post-Operative Period

**Figure 2:**
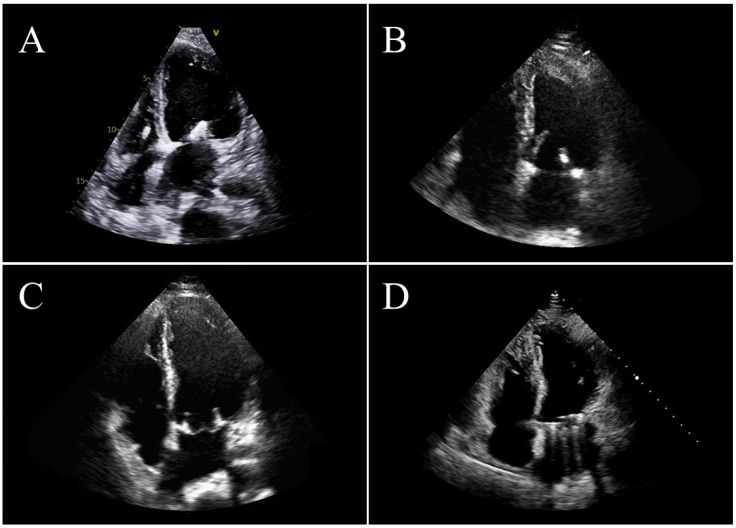
Four patients after mitral valve intervention: (**A**) transcatheter edge-to-edge repair; (**B**) surgical valve repair; (**C**) surgical valve replacement with a biological heart valve; (**D**) surgical valve replacement with a mechanical heart valve.

Mitral valve interventions, including surgical repair/replacement and TEER, require comprehensive post-procedural assessment to ensure optimal patient outcomes (Figure 2). Adequate management of these patients should begin in the early postoperative phase and continue throughout hospitalization. The management has to be tailored to each patient, overseen by a multidisciplinary team. This holistic approach is essential for improving the quality of care and preparing each patient for subsequent rehabilitation phases [37]. Intra-operative assessment by transesophageal echocardiography (TEE) plays a pivotal role in assessing the results in terms of the degree of MR reduction, severity of residual MR, trans-mitral mean gradient, and in identifying complications, which are key components of the decision-making process. In surgical patients, once mitral valve repair is completed, echocardiography plays a crucial role in the immediate post-cardiopulmonary bypass phase. It is essential to assess valve function, determine the need for surgical revision, and guide hemodynamic management [38]. Successful repair was defined as a reduction in MR below a mild level. Any higher regurgitation degree is associated with an increased risk of future reintervention [39]. Additionally, an extensive leaflet coaptation surface is linked to a lower risk of residual regurgitation [40], while the absence of systolic anterior motion (SAM) also indicates an optimal repair outcome. Following mitral valve repair, SAM occurs in approximately 4–10% of cases [41]. This phenomenon results from systolic displacement of the anterior leaflet toward the left ventricular outflow tract, which creates a Venturi effect that can lead to a significant obstruction and a residual regurgitation. The early detection of an SAM through echocardiography is essential for guiding treatment strategies. These may be treated with medical therapy, whereas surgical revision may be required in severe or refractory cases [42]. Additionally, the early assessment of mitral valve gradients is crucial for identifying potential postoperative stenosis, which is often a consequence of an undersized annuloplasty ring [43]. In cases of mitral valve replacement with a prosthetic valve, a comprehensive echocardiographic evaluation should be performed immediately after weaning from cardiopulmonary bypass. This assessment should follow current recommendations for prosthetic valve evaluation, with particular focus on detecting paravalvular leaks and analyzing transvalvular gradients [44]. Echocardiography also plays a crucial role in monitoring ventricular function after the completion of mitral valve surgery. Even in the absence of coronary artery disease, global ventricular dysfunction constitutes a well-documented risk following the repair of a regurgitant mitral valve [45]. Additionally, regional wall motion abnormalities may arise from air embolism or circumflex artery narrowing/occlusion caused by the annuloplasty ring [38]. When percutaneous edge-to-edge repair is performed, TEE is essential throughout the procedure as it guides each step, from trans-septal puncture to clip deployment, thus ensuring an optimal balance between the reduction in regurgitation and the risk of iatrogenic stenosis. Post-procedurally, echocardiography is used to confirm clip stability, to rule out cardiac tamponade, and to assess any residual shunting through a possible iatrogenic septal defect, which is generally not clinically evident after catheter removal [46]. Any residual regurgitation significantly impacts long-term outcomes, thus making precise correction crucial for maximizing procedural benefits and minimizing complications [47]. During the early postoperative period, effective patient management requires an integrated approach combining clinical, biochemical, electrocardiographic, and echocardiographic data to optimize treatment and enable the early detection of complications. Following this procedure, close monitoring of the patient’s status is essential. Blood pressure, heart rate, and any change in clinical symptoms or signs should be closely monitored. Symptoms such as breathlessness, fatigue, or palpitations must be carefully evaluated, as they may indicate an underlying complication. Systemic embolization and arrhythmias should also be promptly identified and appropriately managed to prevent adverse outcomes. Following mitral valve surgery, pacemaker implantation is needed in approximately 4.3% of patients [48]. Continuous electrocardiographic monitoring is essential to detect atrioventricular blocks or pathological pauses, ensuring timely intervention and optimal patient outcomes. Postoperatively, both transthoracic (TTE) and TEE are utilized. Although echocardiographic image quality after surgery may be suboptimal, several studies confirm that both the transthoracic and the transesophageal approach remain an essential diagnostic tool, since both provide critical information that influences clinical decision-making [49]. TTE is a non-invasive and widely accessible tool for routine monitoring. It enables physicians to assess left ventricular function, to evaluate residual MR, and to measure the mitral valve area and gradient. Moreover, TTE is helpful in detecting complications such as pericardial effusion, thrombus formation, and issues related to prosthetic valve function. The transesophageal technique provides clearer and more detailed images, especially in cases involving prosthetic valves or TEER clips. It is particularly useful for assessing leaflet mobility, detecting residual MR, and identifying thrombi. When TTE images are unclear or inconclusive, TEE becomes the preferred imaging method. After surgical or transcatheter mitral valve repair, continuous echocardiographic monitoring is essential to assess the success of the procedure and to detect potential complications. Some degree of MR is common after the procedure, but its severity can impact long-term outcomes. Color Doppler imaging helps to determine the extent of regurgitation, while parameters such as the effective regurgitant orifice area (EROA) and the vena contracta width provide a more precise assessment of its severity. Monitoring mitral valve gradients is crucial to ensure that there are no obstructions after repair. Peak and mean trans-mitral gradients help assess potential narrowing, while pressure half-time (PHT) measurement is useful in estimating the mitral valve area and in detecting possible signs of stenosis. If significant residual MR or stenosis persists, a second intervention, such as a repeat TEER or valve replacement, may be needed. The decision should be based on echocardiographic findings and clinical symptoms. Evaluating overall heart function is essential. LVEF offers insight into systolic function, while left atrial size and pulmonary pressures help predict long-term prognosis. Identifying any signs of diastolic dysfunction is also an important aspect of the postoperative assessment. SAM can occur after mitral valve repair, particularly in patients with a small left ventricular cavity. Early detection is necessary to prevent hemodynamic instability. Pericardial effusions are common after cardiac surgery, while cardiac tamponade is less frequent and more prevalent among patients receiving anticoagulation therapy. As mitral valve surgery typically requires anticoagulation, these patients are at a higher risk of tamponade, a potentially life-threatening condition. TTE or TEE are crucial for detecting and monitoring significant pericardial effusions and thus facilitating timely intervention [50]. Another key aspect of postoperative management is the evaluation and treatment of right ventricular dysfunction, a condition associated with poor prognosis, including increased mortality, stroke, reintubation, and prolonged intensive care unit (ICU) stay [51].

Equally important to the echocardiographic assessment is the functional evaluation, which allows clinicians to determine the patient’s functional capacity both prior to initiating the rehabilitation program and as a final assessment to verify recovery and determine the starting point for any further rehabilitation pathway. This evaluation includes the 6-Minute Walk Test (6MWT), which measures the distance a patient can walk on a flat surface in six minutes and reflects submaximal functional capacity, and the Short Physical Performance Battery (SPPB), a tool used to assess lower extremity function and mobility, consisting of three components: balance tests, gait speed test, and chair stand test.

In conclusion, comprehensive echocardiographic monitoring after mitral valve intervention, together with the assessment of functional capacity, is crucial for the early detection of complications and for optimizing patient outcomes.

## 4. The Role of Rehabilitation During the Post-Operative Period: Phase I Cardiac Rehabilitation

Phase I CR is a critical phase for establishing the basis for recovery after open-heart surgery. It has to start as soon as possible in the ICU, where early mobilization constitutes one of the main components of Phase I rehabilitation, which typically lasts 3 to 7 days [52]. Several studies have shown that early initiation of physical activity on the first or second postoperative day significantly improves patients’ functional outcome [53]. Early mobilization is essential to improve physical status and to reduce the negative effect of bed rest, which includes loss of muscle strength, reduced aerobic capacity, and increased risk of complications [54].

In addition to early mobilization, respiratory muscle training (RMT) is a key component of Phase I rehabilitation [55]. Cardiac surgery often leads to respiratory muscle weakness and reduced lung function secondary to general anesthesia, sternotomy, and cardiopulmonary bypass. RMT, which includes both inspiratory muscle training (IMT) and expiratory muscle training (EMT), has been shown to reduce respiratory impairment (leading to improvements in respiratory muscle strength and overall pulmonary function). For example, research on high-load, long-duration postoperative IMT has demonstrated significant improvements in inspiratory muscle strength and overall lung function [56]. Similarly, a systematic review and meta-analysis evaluating the efficacy of RMT during the early postoperative period of cardiac surgery reported that both IMT and EMT are effective in increasing respiratory muscle strength. Although some studies reported an effect of IMT in improving tidal volume, data on its impact on peak expiratory rate are less conclusive [57].

Another critical aspect of Phase I rehabilitation is the implementation of comprehensive and multidisciplinary advanced recovery protocols. These protocols integrate a variety of interventions, including early mobilization, respiratory training, pain management, and nutritional and psychological support. Advanced recovery programs have the potential to not only improve immediate postoperative outcome, but also to reduce hospital length of stay and readmission rates [20]. Although originally developed for other surgical specialties, these protocols are increasingly being adapted to cardiac surgery, as they offer a holistic approach that also addresses the unique needs of cardiac patients, such as reduced exercise tolerance, psychological distress (e.g., anxiety or depression), the need for medication adherence (e.g., anticoagulation management), and education on lifestyle modifications and cardiovascular risk reduction. These protocols have been shown to reduce perioperative complications and decrease readmission rates [58,59].

In conclusion, phase I CR is essential for recovery after valve surgery or intervention. It improves functional and exercise capacity, physical performance, and muscular strength and reduces physical frailty in the short and medium term [19,60]. Despite strong evidence supporting the benefits of early rehabilitation, challenges remain in ensuring broad participation and optimizing personalized care.

## 5. Cardiac Rehabilitation in Patients After Mitral Valve Intervention: Phase II

Continuing CR programs during phase II, typically conducted in an outpatient setting and lasting 6 to 12 weeks, should be available for all patients undergoing mitral valve surgery or TEER for severe primary regurgitation, as they have been shown to improve short-term functional capacity despite insufficient evidence regarding the impact on primary outcomes such as mortality, hospitalization, and QoL in this patient population [13]. Nevertheless, given the well-established benefits of physical exercise in patients with cardiovascular disease, similar advantages may be expected in this group, particularly in those with a postoperative course complicated by heart failure [12]. A potential barrier to referral for rehabilitation may be concerns regarding the possible adverse effects. However, considering the documented benefits of early rehabilitation in the immediate postoperative period, a phase of particular vulnerability, further advantages can be expected in the later stages. Moreover, extending the rehabilitation program would enable long-term patient management and education, facilitating the early detection of potential postoperative complications or arrhythmias [13,61].

**Figure 3:**
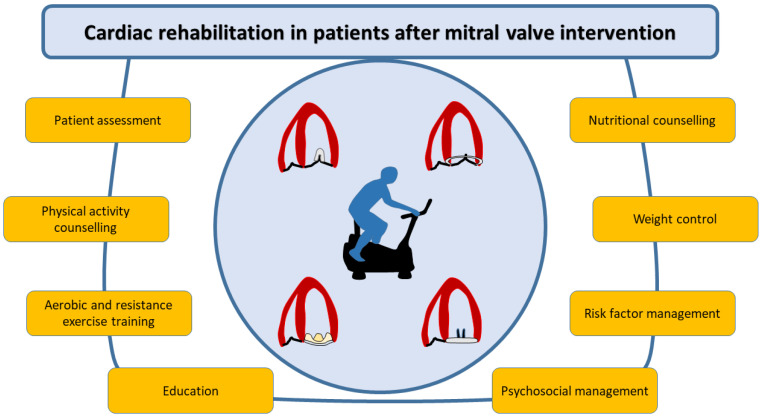
Core components of cardiac rehabilitation in patients after mitral valve intervention.

This rehabilitation phase focuses on monitoring the progression of individualized physical exercise based on clinical assessment (Figure 3). Initial evaluation should include a comprehensive medical history, considering comorbidities, perioperative heart failure, postoperative pain management, and risk stratification and functional capacity assessment [62]. Assessing preoperative physical activity and conducting a thorough physical examination are crucial for evaluating clinical status, wound healing, venous thromboembolism, pleural effusion, diaphragmatic paralysis, and exercise limitations. Frailty should be identified using a dedicated scoring system. Reassessment should include laboratory tests (CBC, iron profile, renal and liver function, glucose, and lipid profiles), ECG for rhythm abnormalities, and echocardiography to evaluate residual atrial septal defect (if percutaneous repair was performed), trans-mitral gradient, residual MR, systolic and diastolic function, and pericardial effusion [63]. Patients should be educated on thromboembolism prevention, drug interactions, and lifelong anticoagulation when indicated. Anticoagulation is required for three months in bioprosthesis or mitral repairs with prosthetic annuloplasty rings and lifelong for patients with mechanical prostheses. Self-management may contribute to reducing International Normalized Ratio (INR) variability, a key predictor of survival after valve replacement. Endocarditis prophylaxis education is also essential [12], and for percutaneous procedures patients should be monitored for at least six months post-procedure.

Dietary counseling is essential for promoting a healthy diet to reduce cardiovascular risk and, in anticoagulated patients, to minimize interactions with vitamin K-rich foods or other medications. Nutritional assessment is particularly important in elderly patients to determine the need for micronutrient (vitamins, minerals, iron, folate) or macronutrient (protein, fluid) supplementation to prevent malnutrition. In cases of heart failure, education on fluid restriction, sodium reduction, and weight management is crucial. If clinical compensation improves during rehabilitation, diuretic therapy should be adjusted accordingly. A multimodal approach should include not only education and nutritional counseling but also psychological support. Psychosocial risk factor assessment should cover aspects like socio-economic status, lack of support, stress, mental health issues (such as depression and anxiety), and cognitive impairment. The two-step evaluation in CR involves initially asking about these factors and then using standardized questionnaires, like HeartQoL (a validated questionnaire designed to assess health-related quality of life) or Hospital Anxiety and Depression Scale (HADS, a self-assessment scale commonly used in hospital settings to detect states of anxiety and depression), for a more comprehensive assessment [63].

**Figure 4:**
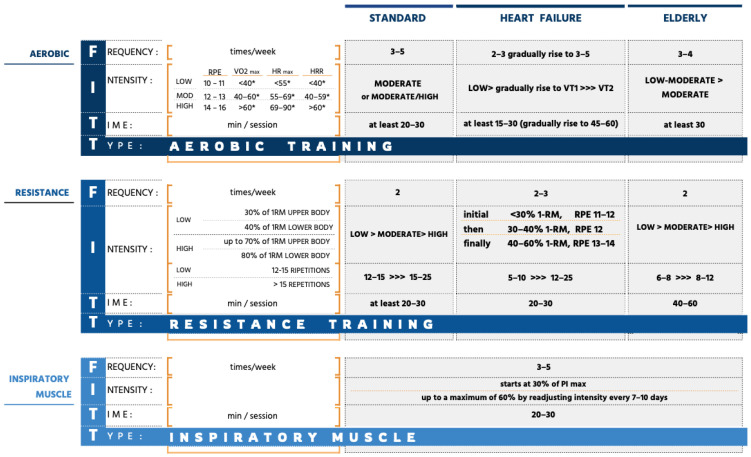
Training after mitral valve intervention. * Expressed as percentage. HR max, maximal heart rate; HRR, heart rate reserve; RPE, ratings of perceived exertion; VO2, peak oxygen uptake; VT1, first ventilatory threshold; VT2, secondary ventilatory threshold; 1-RM, one repetition maximum.

All patients should receive guidance on physical activity, considering both wound healing and exercise capacity. Inpatient and/or outpatient exercise training should begin immediately after discharge and be individualized based on clinical conditions, ventricular function, and the results of an early symptom-limited functional test, followed by a maximal exercise test [63]. CR can enhance the hemodynamic benefits of valve correction by improving exercise capacity, particularly in patients who do not experience spontaneous peak VO_2_ improvement [64]. Studies have shown its effectiveness in increasing peak VO_2_, regardless of age, sex, valve surgery type, or concurrent bypass surgery [64]. However, post-valve surgery patients generally have lower functional capacity than those undergoing bypass surgery. Among post-valve patients, mitral valve replacement patients demonstrate reduced exercise tolerance compared to aortic valve surgery patients, especially in the presence of residual pulmonary hypertension [12]. Research on exercise prescription for patients with PMR undergoing valve surgery is limited, with even fewer recommendations available for those treated with percutaneous corrective procedures. While postoperative functional capacity is largely influenced by preoperative risk, the benefits of physical training appear to be independent of both preoperative risk and the type of intervention. When designing an exercise program, factors beyond the corrective technique (surgical vs. percutaneous) should be considered, as the technique itself does not impact the functional gains achieved through rehabilitation [65]. Although high-risk patients have lower postoperative functional capacity, exercise benefits remain comparable, with differences primarily attributable to baseline status. In percutaneous correction, reduced capacity is mainly due to age and comorbidities, not the procedure itself. Therefore, a comprehensive patient assessment and a personalized exercise program are essential for optimizing rehabilitation outcomes. The standard supervised training program should follow the FITT (Frequency, Intensity, Time/Duration, and Type/Mode of Exercise) prescription model (Figure 4). Exercise dose, frequency, duration, and intensity must be tailored to each patient based on functional assessments, such as cardiopulmonary exercise testing [63]. Properly determining exercise intensity before initiating a training program is crucial to ensuring both the effectiveness and safety of CR [66]. Currently, no standardized FITT-based guidelines exist for post-mitral valve surgery patients. However, a core rehabilitation program should include low-to-moderate intensity aerobic training (≥3×/week, 20–30 min/session, ideally 45–60 min), complemented by resistance training (2×/week, starting at low intensity and progressing to moderate, targeting major muscle groups). Upper-body-strength training can begin once the chest is stable, typically six weeks post-surgery. Additionally, balance training (2–3×/week, 10–15 min/session) and daily respiratory muscle training (15 min) are recommended, particularly in patients with prolonged postoperative mechanical ventilation, respiratory comorbidities, or concomitant heart failure [63]. Guidelines for rehabilitation after TEER are scarce, likely due to concerns about early clip detachment. However, CR can also enhance functional capacity in this population by optimizing peripheral oxygen extraction post-intervention [67]. A recent retrospective analysis assessed the effects of inpatient CR within two months of percutaneous mitral valve repair. Patients followed a standardized program including aerobic and resistance training, inspiratory muscle training, and educational and psychosocial support. To prevent blood pressure peaks and ensure clip stability, training was conducted at low intensity with continuous monitoring. The study confirmed that multimodal rehabilitation was both safe (no device-related complications) and effective in improving functional capacity [27]. Since percutaneous intervention primarily targets older, comorbid patients, physical training should be individualized based on age, frailty, and heart failure status. In elderly patients, rehabilitation should emphasize prolonged endurance exercise, complemented by strength training, calisthenics, respiratory exercises, and varied activities such as outdoor walking, gymnastics, and aquatic therapy. Training intensity should remain moderate, guided by preoperative assessments, rehabilitation progress, and postoperative reassessments [63].

## 6. Cardiac Rehabilitation in Patients After Mitral Valve Intervention: Phase III

The surgical journey extends beyond Phase II into Phase III rehabilitation, a long-term maintenance phase that may last from several months to lifelong. This phase focuses on sustained recovery and the prevention of recurrent cardiovascular events. It encourages sustained lifestyle changes, improves physical fitness and heart health, reduces the risk of recurrence, promotes independence and self-monitoring, and supports psychological well-being.

This phase includes sustained physical training—aerobic, strength, and respiratory exercises—along with psychological support and educational sessions, delivered through outpatient follow-ups or remote tele-rehabilitation. Tele-rehabilitation has already shown benefits in preoperative assessments for cardiac surgery candidates. In phase III, digital health services enable remote monitoring and asynchronous rehabilitation sessions, reducing patient costs (e.g., transportation), optimizing clinical resources, and enhancing adherence to rehabilitation programs [68].

## 7. Tailored Management in Clinical Practice

**Figure 5:**
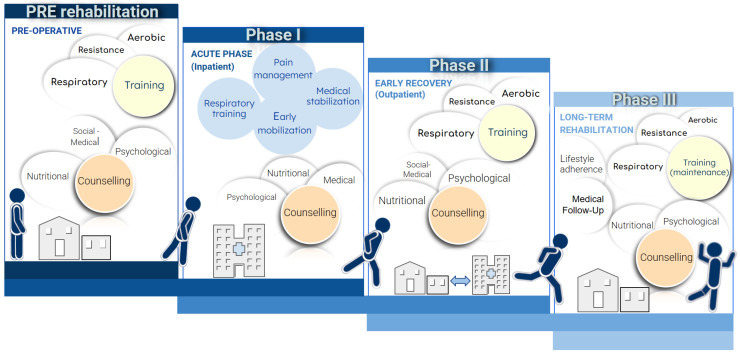
Patient journey with core components of cardiac rehabilitation.

Rehabilitation for patients undergoing surgery or TEER for PMR should follow a multidisciplinary approach, optimizing the clinical conditions before, during, and after the procedure. This structured patient journey aims to maximize functional recovery and aligns with the Enhanced Recovery After Surgery (ERAS) protocol, a validated strategy in general surgery now gaining traction in cardiac surgery and mitral valve disease (Figure 5) [68].

Prehabilitation, or rehabilitation initiated prior to surgery, has been shown to improve outcomes in ischemic heart disease and general surgery patients (see first box in Figure 5). While data on valvular heart disease remain limited due to concerns about potential hemodynamic stress from exercise, emerging evidence suggests that prehabilitation is not only safe but can also enhance functional capacity, prevent deterioration, and shorten hospital stays. In MR, ejection fraction may underestimate systolic dysfunction and fail to reflect ventricular performance accurately. Exercise-induced tachycardia shortens diastolic time, reduces regurgitant volume, and may improve cardiac output without significantly increasing pulmonary congestion [69]. Early preoperative assessment is crucial for identifying patients—especially those with frailty—who would benefit most from prehabilitation. Frailty, characterized by a decline in physiological reserve across clinical, physical, cognitive, and social domains, is associated with higher postoperative complications, prolonged ventilation, extended hospital stays, reduced QoL, increased healthcare costs, and higher long-term mortality [70]. Early detection allows for targeted interventions to optimize functional capacity and reduce the risk of adverse events. Notably, patients who completed a home-based prehabilitation program showed significant improvements in functional ability and exercise capacity, reinforcing the importance of integrating frailty assessments into surgical risk stratification [71]. Prehabilitation programs typically include aerobic endurance training, respiratory muscle strengthening, chest expansion exercises, and ventilatory work. IMT enhances respiratory muscle function, improving gas exchange, reducing postoperative pulmonary complications, and increasing respiratory reserve, leading to better lung function and improved 6 min walk test (6MWT) performance [72]. Additionally, resistance training, when combined with aerobic exercise and emphasizing both pulmonary and peripheral muscle conditioning, can further improve physical fitness, participation in rehabilitation, and overall QoL in patients undergoing valve surgery [73]. The period following valve cardiac surgery and immediately after discharge, corresponding to Phase I and II (see the second and third boxes in Figure 5), represents a particularly critical stage, marked by heightened vulnerability to complications and psychophysical stress, increasing the risk of hospital readmission. Moreover, physical activity levels after surgery remain significantly lower compared to the general population, with almost half of patients failing to meet the international recommendations. This highlights the importance of a structured CR program within 6–12 months after surgery to support recovery and minimize the risk of readmission. Such a program serves as an ideal setting to address both critical phases: an early, intensive intervention targeting the management of medical complications and preventing short-term hospital readmissions (Phases I and II), followed by a long-term strategy focused on restoring physical function and psychological well-being (Phase III) [74]. Early rehabilitation during Phase I can be facilitated by selecting minimally invasive surgical techniques that reduce cardiopulmonary bypass time, alongside fast-track anesthesia protocols featuring low-dose opioids and regional analgesia. This approach enables early extubation, shortens mechanical ventilation duration, and allows the initiation of physiotherapy within hours of surgery [68]. A recent review examined the impact of CR, consisting of physical exercise with or without a psychoeducational component, after heart valve surgery. Exercise can provide significant benefits for this category of patients, similar to those observed in other groups, by improving hemodynamic and physiological parameters and short-term exercise capacity, potentially reducing hospitalizations and enhancing QoL [13]. Psychological support should also be an integral part of rehabilitation in every phase, starting from pre-rehabilitation and continuing after surgery. Patients may experience post-traumatic stress from an acute event or anxiety and depression due to eagerness to resume daily life too quickly. Post-surgical patients may also struggle with sternotomy-related pain and sleep disturbances. In older patients undergoing percutaneous repair, cognitive decline, such as memory deficits or slower processing speed, may further impact QoL, self-care, and treatment adherence. The various multimodal components of the rehabilitation process should continue in the long term during Phase III (see fourth box in Figure 5), which is crucial for preventing a recurrence of adverse cardiovascular events. This phase can be supported by digital health systems. Although studies have shown that telerehabilitation is as effective as center-based CR in improving clinical outcomes and health-related QoL in patients after myocardial infarction, revascularization, or heart failure, evidence remains limited for post-valve surgery patients [75]. Early experiences suggest that digital health services improve outcomes, increase patient and operator satisfaction, and promote high patient engagement, while also reducing healthcare costs. Offering asynchronous sessions provides benefits by eliminating the need for additional outpatient resources and making it easier to follow education, self-monitoring, motivational feedback, and therapy programs. This approach enhances compliance and leverages widely accessible digital tools, aligning with the growing trend of digitalization [68]. However, further studies are needed to determine whether the benefits of digital/telehealth rehabilitation are sustained over the long term and whether they extend to other cardiac populations, such as post-valve surgery patients, and particularly those undergoing percutaneous interventions.

## 8. Conclusions

CR is fundamental for optimizing patient recovery after surgical or percutaneous intervention of the mitral valve, but its implementation remains limited in clinical practice.

A structured patient journey, beginning with prehabilitation and continuing through all rehabilitation phases, is crucial for achieving the best functional recovery. This pathway should be individualized based on patient characteristics, including age, comorbidities, and baseline functional status. A multimodal approach, including exercise training, psychological and educational support, nutritional counseling, and digital health solutions, enhances outcomes and QoL.

Telerehabilitation has shown comparable effectiveness to center-based programs, but further research is needed to confirm long-term benefits, particularly in post-valve surgery patients. Novel valve repair techniques, including percutaneous procedures, allow for earlier rehabilitation initiation by avoiding sternotomy, but patients are often older and have more comorbidities.

Future CR programs should explore alternative delivery models, including home-based programs, to expand access and improve adherence. High-quality trials are needed to refine rehabilitation strategies and ensure long-term benefits across diverse cardiac populations.

## Figures and Tables

**Table 1:** The impact of cardiac rehabilitation in patients after CABG and aortic and mitral valve interventions.

Type of Intervention	Evidence of CR Efficacy	Observed Outcomes	Limitations
CABG	Strong and well-established	Improved exercise capacity, QoL, reduced mortality, and rehospitalization	High-quality data from multiple RCTs and meta-analyses
Aortic Valve Surgery	Moderate evidence	Improved functional capacity, QoL, and reduced rehospitalization	Limited controlled trials; heterogeneity in patient profiles
Mitral Valve Surgery	Inconclusive evidence	Some benefits in aerobic fitness and QoL, especially with minimally invasive approaches	Limited and heterogeneous data; few high-quality studies
Mitral Valve TEER	Limited and emerging evidence	Improved endurance, handgrip strength, balance, and mobility (especially in elderly/frail patients)	Based mainly on case reports and small retrospective studies; lack of RCTs

CABG, Coronary artery bypass grafting; CR, cardiac rehabilitation; QoL, quality of life; RCT, randomized controlled trial; TEER, transcatheter edge-to-edge repair.

## Data Availability

Not applicable.
